# Clinician-Administered PTSD Scale for DSM-5,child and adolescent version: Clinical characteristics of paediatric population

**DOI:** 10.1192/j.eurpsy.2023.1549

**Published:** 2023-07-19

**Authors:** N. Kouki, S. Bourgou, H. Rezgui, A. Naffet, M. Hamdoun, A. Belhadj

**Affiliations:** 1 Child an adolescent psychiatry; 2 Child and adolescent psychiatry, Mongi slim; 3 Forensic, charles nicolle, Tunis, Tunisia

## Abstract

**Introduction:**

Posttraumatic stress disorder in the paediatric population has clinical features. The Clinician-Administered PTSD Scale for DSM-5,child and adolescent version (CAPS-CA-5) is the gold standard in positive diagnosis

**Objectives:**

The objectives of our work was to study the clinical characteristics of the paediatric population with the diagnosis of PTSD.

**Methods:**

This is a descriptive cross-sectional study conducted in the child psychiatry department of Mongi Slim Hospital and the forensic medicine department of Charles-Nicolle Hospital, among children older than seven years who were exposed to a potentially traumatic event at least one month before. We made clinical assessment for PTSD using CAPS-CA-5 which is currently being validated in Tunisian dialect. Then We investigated the clinical characteristics of PTSD according to age, gender, history, and event specifics.

**Results:**

We conducted our study with 150 patients . The diagnosis of PTSD according to DSM 5 criteria was retained in 56.2% of patients (N=80).

The average age was 12.4 years with extremes ranging from 7 to 17 years. We noted a female predominance at 58.8% (n=47)

Male gender was significantly associated with persistent avoidance (p=0.03). Sexual assault was significantly associated with the severity of flashback symptoms (p<10-3) and reckless and self-destructive behaviors (p<10-3) and with the frequency of dissociative symptoms (p<10-3).

We also noted in our work that dissociative symptoms were significantly more frequent in victims with no personal psychiatric history with a p value of 0.021.

In our population, we found a predominance of hypervigilance and a greater severity of exaggerated startle reactions in the absence of a family psychiatric history with a p value of 0.048 and 0.008 respectively.

We noted a significant predominance of exaggerated startle reactions in relation to the absence of exposure to previous traumatic events with a p equal to 0.043

**Conclusions:**

The specificities identified in relation to the child should be taken into consideration during further evaluations and further analysis in the general population.

**Disclosure of Interest:**

None Declared

